# On a two-truths phenomenon in spectral graph clustering

**DOI:** 10.1073/pnas.1814462116

**Published:** 2019-03-08

**Authors:** Carey E. Priebe, Youngser Park, Joshua T. Vogelstein, John M. Conroy, Vince Lyzinski, Minh Tang, Avanti Athreya, Joshua Cape, Eric Bridgeford

**Affiliations:** ^a^Department of Applied Mathematics and Statistics, Johns Hopkins University, Baltimore, MD 21218;; ^b^Center for Imaging Science, Johns Hopkins University, Baltimore, MD 21218;; ^c^Human Language Technology Center of Excellence, Johns Hopkins University, Baltimore, MD 21218;; ^d^Department of Biomedical Engineering, Johns Hopkins University, Baltimore, MD 21218;; ^e^Institute for Defense Analyses, Center for Computing Sciences, Bowie, MD 20715;; ^f^Department of Mathematics and Statistics, University of Massachusetts, Amherst, MA 01003;; ^g^Department of Biostatistics, Johns Hopkins University, Baltimore, MD 21218

**Keywords:** spectral embedding, spectral clustering, graph, network, connectome

## Abstract

Clustering is concerned with coherently grouping observations without any explicit concept of true groupings. Spectral graph clustering—clustering the vertices of a graph based on their spectral embedding—is commonly approached via *K*-means (or, more generally, Gaussian mixture model) clustering composed with either Laplacian spectral embedding (LSE) or adjacency spectral embedding (ASE). Recent theoretical results provide deeper understanding of the problem and solutions and lead us to a “two-truths” LSE vs. ASE spectral graph clustering phenomenon convincingly illustrated here via a diffusion MRI connectome dataset: The different embedding methods yield different clustering results, with LSE capturing left hemisphere/right hemisphere affinity structure and ASE capturing gray matter/white matter core–periphery structure.

## Significance

Spectral graph clustering—clustering the vertices of a graph based on their spectral embedding—is of significant current interest, finding applications throughout the sciences. But as with clustering in general, what a particular methodology identifies as “clusters” is defined (explicitly, or, more often, implicitly) by the clustering algorithm itself. We provide a clear and concise demonstration of a “two-truths” phenomenon for spectral graph clustering in which the first step—spectral embedding—is either Laplacian spectral embedding, wherein one decomposes the normalized Laplacian of the adjacency matrix, or adjacency spectral embedding given by a decomposition of the adjacency matrix itself. The two resulting clustering methods identify fundamentally different (true and meaningful) structure.

The purpose of this paper is to cogently present a “two-truths” phenomenon in spectral graph clustering, to understand this phenomenon from a theoretical and methodological perspective, and to demonstrate the phenomenon in a real-data case consisting of multiple graphs each with multiple categorical vertex class labels.

A graph or network consists of a collection of vertices or nodes V representing n entities together with edges or links E representing the observed subset of the n2 possible pairwise relationships between these entities. Graph clustering, often associated with the concept of “community detection,” is concerned with partitioning the vertices into coherent groups or clusters. By its very nature, such a partitioning must be based on connectivity patterns.

**Fig. 1.**
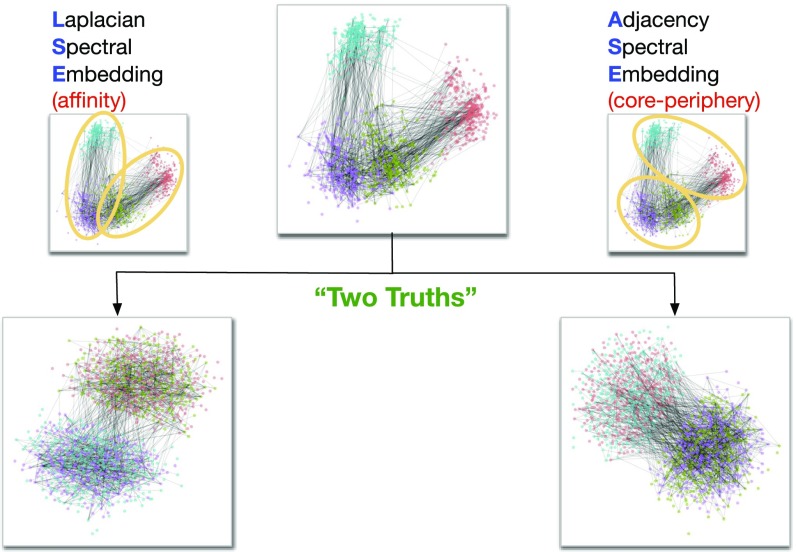
A two-truths graph (connectome) depicting connectivity structure such that one grouping of the vertices yields affinity structure (e.g., left hemisphere/right hemisphere) and the other grouping yields core–periphery structure (e.g., gray matter/white matter). (*Top Center*) The graph with four vertex colors. (*Top Left* and *Top Right*) LSE groups one way and ASE groups another way. (*Bottom Left*) The LSE truth is two densely connected groups, with sparse interconnectivity between them (affinity structure). (*Bottom Right*) The ASE truth is one densely connected group, with sparse interconnectivity between it and the other group and sparse interconnectivity within the other group (core–periphery structure). This paper demonstrates the two-truths phenomenon illustrated here—that LSE and ASE find fundamentally different but equally meaningful network structure—via theory, simulation, and real data analysis.

It is often the case that practitioners cluster the vertices of a graph—say, via K-means clustering composed with Laplacian spectral embedding—and pronounce the method as having performed either well or poorly based on whether the resulting clusters correspond well or poorly with some known or preconceived notion of “correct” clustering. Indeed, such a procedure may be used to compare two clustering methods and to pronounce that one works better (on the particular data under consideration). However, clustering is inherently ill-defined, as there may be multiple meaningful groupings, and two clustering methods that perform differently with respect to one notion of truth may in fact be identifying inherently different, but perhaps complementary, underlying structure. With respect to graph clustering, ref. 1 shows that there can be no algorithm that is optimal for all possible community detection tasks (Fig. 1).

We compare and contrast Laplacian and adjacency spectral embedding as the first step in spectral graph clustering and demonstrate that the two methods, and the two resulting clusterings, identify different—but both meaningful—graph structure. We trust that this simple, clear explication will contribute to an awareness that connectivity-based structure discovery via spectral graph clustering should consider both Laplacian and adjacency spectral embedding and the development of new methodologies based on this awareness.

## Spectral Graph Clustering

Given a simple graph G=(V,E) on n vertices, consider the associated n×n adjacency matrix A in which $A_{ij}$ = 0 or 1 encoding whether vertices i and j in V share an edge (i,j) in E. For our simple undirected, unweighted, loopless case, A is binary with $A_{ij}\in \left\{0,1\right\}$, symmetric with $A=A^{⊤}$, and hollow with $diag\left(A\right)=\vec{0}$.

The first step of spectral graph clustering (2, 3) involves embedding the graph into Euclidean space via an eigendecomposition. We consider two options: Laplacian spectral embedding (LSE), wherein we decompose the normalized Laplacian of the adjacency matrix, and adjacency spectral embedding (ASE) given by a decomposition of the adjacency matrix itself. With target dimension d, either spectral embedding method produces n points in $R^{d}$, denoted by the n×d matrix X. ASE employs the eigendecomposition to represent the adjacency matrix via $A=USU^{⊤}$ and chooses the top d eigenvalues by magnitude and their associated vectors to embed the graph via the scaled eigenvectors $U_{d}|S_{d}{|}^{1/2}$. Similarly, LSE embeds the graph via the top scaled eigenvectors of the normalized Laplacian $L\left(A\right)=D^{-1/2}AD^{-1/2}$, where D is the diagonal matrix of vertex degrees. In either case, each vertex is mapped to the corresponding row of $X=U_{d}|S_{d}{|}^{1/2}$.

Spectral graph clustering concludes via classical Euclidean clustering of the rows of X. As described below, central limit theorems for spectral embedding of the (sufficiently dense) stochastic block model via either LSE or ASE suggest Gaussian mixture modeling (GMM) for this clustering step. Thus, we consider spectral graph clustering to be GMM composed with LSE or ASE:

GMM ○ {LSE,ASE}.

## Stochastic Block Model

The random graph model we use to illustrate our phenomenon is the stochastic block model (SBM), introduced in ref. 4. This model is parameterized by (*i*) a block membership probability vector $\vec{\pi }={\left[\pi_{1},\ldots ,\pi_{K}\right]}^{⊤}$ in the unit simplex and (*ii*) a symmetric K×K block connectivity probability matrix B with entries in [0,1] governing the probability of an edge between vertices given their block memberships. Use of the SBM is ubiquitous in theoretical, methodological, and practical graph investigations, and SBMs have been shown to be universal approximators for exchangeable random graphs (5).

For sufficiently dense graphs, both LSE and ASE have a central limit theorem (6–8) demonstrating that, for large n, embedding via the top d eigenvectors from a rank d K-block SBM (d≡rank(B)≤K) yields n points in $R^{d}$ behaving approximately as a random sample from a mixture of K Gaussians. That is, given that the ith vertex belongs to block k, the ith row of $X=U_{d}{\left|S_{d}\right|}^{1/2}$ will be approximately distributed as a multivariate normal with parameters specific to block k, $X_{i}∼MVN\left(\mu_{k},\Sigma_{k}\right)$. The structure of the covariance matrices suggests that the GMM is called for, as an appropriate generalization of K-means clustering. Therefore, GMM(X) via maximum likelihood will produce mixture parameter estimates and associated asymptotically perfect clustering, using either LSE or ASE. For finite n, however, LSE and ASE yield different clustering performance, and neither one dominates the other.

We make significant conceptual use of the positive definite two-block SBM (K=2), with

$$B=\left[\begin{array}{cc} B_{11} & B_{12}\\ B_{21} & B_{22} \end{array}\right]=\left[\begin{array}{cc} a & b\\ b & c \end{array}\right]$$

which henceforth we abbreviate as B=[a,b;b,c]. In this simple setting, two general/generic cases present themselves: affinity and core–periphery.

### Affinity: a,c≫b.

An SBM with B=[a,b;b,c] is said to exhibit affinity structure if each of the two blocks has a relatively high within-block connectivity probability compared with the between-block connectivity probability.

### Core-periphery: a≫b,c.

An SBM with B=[a,b;b,c] is said to exhibit core–periphery structure if one of the two blocks has a relatively high within-block connectivity probability compared with both the other block’s within-block connectivity probability and the between-block connectivity probability.

The relative performance of LSE and ASE for these two cases provides the foundation for our analyses. Informally, LSE outperforms ASE for affinity, and ASE is the better choice for core–periphery. We make this clustering performance assessment analytically precise via Chernoff information, and we demonstrate this in practice via the adjusted Rand index.

## Clustering Performance Assessment

We consider two approaches to assessing the performance of a given clustering, defined to be a partition of [n]≡{1,…,n} into a disjoint union of K partition cells or clusters. For our purposes—demonstrating a two-truths phenomenon in LSE vs. ASE spectral graph clustering—we consider the case in which there is a “true” or meaningful clustering of the vertices against which we can assess performance, but we emphasize that in practice such a truth is neither known nor necessarily unique.

### Chernoff Information.

Comparing and contrasting the relative performance of LSE vs. ASE via the concept of Chernoff information (9, 10), in the context of their respective central limit theorems (CLTs), provides a limit theorem notion of superiority. Thus, in the SBM, we allude to the GMM provided by the CLT for either LSE or ASE.

The Chernoff information between two distributions is the exponential rate at which the decision-theoretic Bayes error decreases as a function of sample size. In the two-block SBM, with the true clustering of the vertices given by the block memberships, we are interested in the large-sample optimal error rate for recovering the underlying block memberships after the spectral embedding step has been carried out. Thus, we require the Chernoff information $C\left(F_{1},F_{2}\right)$ when $F_{1}=MVN\left(\mu_{1},\Sigma_{1}\right)$ and $F_{2}=MVN\left(\mu_{2},\Sigma_{2}\right)$ are multivariate normals. Letting $\Sigma_{t}=t\Sigma_{1}+\left(1-t\right)\Sigma_{2}$ and

$$\begin{array}{ll} h\left(t;F_{1},F_{2}\right)= & \frac{t\left(1-t\right)}{2}{\left(\mu_{1}-\mu_{2}\right)}^{⊤}\Sigma_{t}^{-1}\left(\mu_{1}-\mu_{2}\right)\\ & +\frac{1}{2}\log\frac{\left|\Sigma_{t}\right|}{|\Sigma_{1}{|}^{t}|\Sigma_{2}{|}^{1-t}}, \end{array}$$

we have

$$\rho_{F_{1},F_{2}}=\underset{t\in \left(0,1\right)}{\sup}h\left(t;F_{1},F_{2}\right).$$

This provides both $\rho_{L}$ and $\rho_{A}$ when using the large-sample GMM parameters for $F_{1},F_{2}$ obtained from the LSE and ASE embeddings, respectively, for a particular two-block SBM distribution (defined by its block membership probability vector $\vec{\pi }$ and block connectivity probability matrix B). We make use of the Chernoff ratio $\rho =\rho_{A}/\rho_{L}$; ρ>1 implies ASE is preferred while ρ<1 implies LSE is preferred. (Recall that as the Chernoff information increases, the large-sample optimal error rate decreases.) Chernoff analysis in the two-block SBM demonstrates that, in general, LSE is preferred for affinity while ASE is preferred for core–periphery (7, 11).

### Adjusted Rand Index.

In practice, we wish to empirically assess the performance of a particular clustering algorithm on a given graph. There are numerous cluster assessment criteria available in the literature: the Rand index (RI) (12), normalized mutual information (NMI) (13), variation of information (VI) (14), Jaccard (15), etc. These are typically used to compare either an empirical clustering against a “truth” or two separate empirical clusterings. For concreteness, we consider the well-known adjusted Rand index (ARI), popular in machine learning, which normalizes the RI so that expected chance performance is zero: The ARI is the adjusted-for-chance probability that two partitions of a collection of data points will agree for a randomly chosen pair of data points, putting the pair into the same partition cell in both clusterings or splitting the pair into different cells in both clusterings. (Our empirical connectome results are essentially unchanged when using other cluster assessment criteria.)

In the context of spectral clustering via GMM○{LSE,ASE}, we consider $C_{LSE}$ and $C_{ASE}$ to be the two clusterings of the vertices of a given graph. Then ARI($C_{LSE},C_{ASE}$) assesses their agreement: ARI($C_{LSE},C_{ASE}$) =1 implies that the two clusterings are identical; ARI($C_{LSE},C_{ASE}$) ≈ 0 implies that the two spectral embedding methods are “operationally orthogonal.” (Significance is assessed via permutation testing.)

In the context of two truths, we consider $C_{1}$ and $C_{2}$ to be two known true or meaningful clusterings of the vertices. Then, with $C_{SE}$ being either $C_{LSE}$ or $C_{ASE}$, ARI($C_{SE},C_{1}$) ≫ ARI($C_{SE},C_{2}$) implies that the spectral embedding method under consideration is more adept at discovering truth $C_{1}$ than truth $C_{2}$. Analogous to the theoretical Chernoff analysis, ARI simulation studies in the two-block SBM demonstrate that, in general, LSE is preferred for affinity while ASE is preferred for core–periphery.

## Model Selection × 2

To perform the spectral graph clustering GMM○ {LSE,ASE} in practice, we must address two inherent model selection problems: We must choose the embedding dimension ($\hat{d}$) and the number of clusters ($\hat{K}$).

### SBM vs. Network Histogram.

If the SBM were actually true, then as n→∞ any reasonable procedure for estimating the singular value decomposition (SVD) rank would yield a consistent estimator $\hat{d}\to d$ and any reasonable procedure for estimating the number of clusters would yield a consistent estimator $\hat{K}\to K$. Critically, the universal approximation result of ref. 5 shows that SBMs provide a principled “network histogram” model even without the assumption that the SBM with some fixed (d,K) actually holds. Thus, practical model selection for spectral graph clustering is concerned with choosing ($\hat{d},\hat{K}$) to provide a useful approximation.

The bias–variance tradeoff demonstrates that any quest for a universally optimal methodology for choosing the “best” dimension and number of clusters, in general, for finite n, is a losing proposition. Even for a low-rank model, subsequent inference may be optimized by choosing a dimension smaller than the true signal dimension, and even for a mixture of K Gaussians, inference performance may be optimized by choosing a number of clusters smaller than the true cluster complexity. In the case of semiparametric SBM fitting, wherein low-rank and finite mixtures are used as a practical modeling convenience as opposed to a believed true model, and one presumes that both $\hat{d}$ and $\hat{K}$ will tend to infinity as n→∞, these bias–variance tradeoff considerations are exacerbated.

For $\hat{d}$ and $\hat{K}$ below, we make principled methodological choices for simplicity and concreteness, but make no claim that these are best in general or even for the connectome data considered herein. Nevertheless, one must choose an embedding dimension and a mixture complexity, and thus we proceed.

### Choosing the Embedding Dimension $\hat{d}$.

A ubiquitous and principled general methodology for choosing the number of dimensions in eigendecompositions and SVDs (e.g., principal components analysis, factor analysis, spectral embedding, etc.) is to examine the so-called scree plot and look for “elbows” defining the cutoff between the top (signal) dimensions and the noise dimensions. There are a plethora of variations for automating this singular value thresholding (SVT); section 2.8 of ref. 16 provides a comprehensive discussion in the context of principal components, and ref. 17 provides a theoretically justified (but perhaps practically suspect, for small n) universal SVT. We consider the profile-likelihood SVT method of ref. 18. Given $A=USU^{⊤}$ (for either LSE or ASE) the singular values S are used to choose the embedding dimension $\hat{d}$ via

$$\hat{d}=\arg\underset{d}{\max}ProfileLikelihood_{S}\left(d\right),$$

where $ProfileLikelihood_{S}\left(d\right)$ provides a definition for the magnitude of the “gap” after the first d singular values.

### Choosing the Number of Clusters $\hat{K}$.

Choosing the number of clusters in Gaussian mixture models is most often addressed by maximizing a fitness criterion penalized by model complexity. Common approaches include the Akaike information criterion (AIC) (19), the Bayesian information criterion (BIC) (20), minimum description length (MDL) (21), etc. We consider penalized likelihood via the BIC (22). Given n points in $R^{d}$ represented by $X=U_{d}{\left|S_{d}\right|}^{1/2}$ (obtained via either LSE or ASE) and letting $\theta_{K}$ represent the GMM parameter vector whose dimension $dim\left(\theta_{K}\right)$ is a function of the data dimension d, the mixture complexity $\hat{K}$ is chosen via

$$\hat{K}=\arg\underset{K}{\max}PenalizedLikelihood_{X}\left({\hat{\theta }}_{K}\right),$$

where $PenalizedLikelihood_{X}\left({\hat{\theta }}_{K}\right)$ is twice the log-likelihood of the data X evaluated at the GMM with mixture parameter estimate ${\hat{\theta }}_{K}$ penalized by $dim\left(\theta_{K}\right)\cdot \ln\;n$. For spectral clustering, we use the BIC for $\hat{K}$ after spectral embedding, so $X\in R^{\hat{d}}$ with $\hat{d}$ chosen as above.

## Connectome Data

**Fig. 2.**
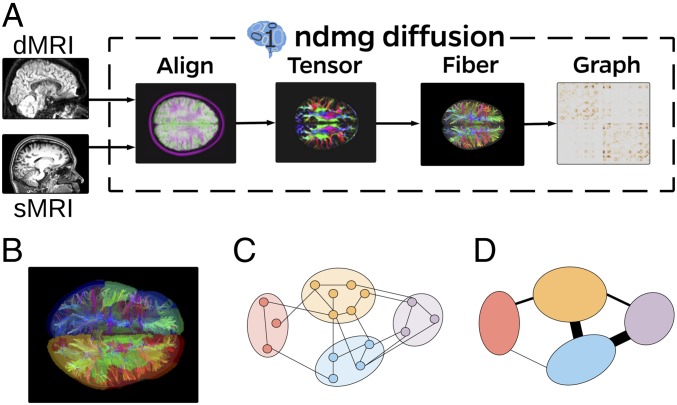
Connectome data generation. (*A*) The pipeline. (*B*) Voxels and regions in tractography map. (*C*) Voxels and edges. (*D*) Contraction yields vertices and edges. The output is diffusion MRI graphs on ≈1 million vertices. Spatial vertex contraction yields graphs on ≈70,000 vertices from which we extract largest connected components of ≈40,000 vertices with {Left,Right} and {Gray,White} labels for each vertex. Fig. 1 depicts (a subsample from) one such graph.

We consider for illustration a diffusion MRI dataset consisting of 114 connectomes (57 subjects, two scans each) with 72,783 vertices each and both left/right/other hemispheric and gray/white/other tissue attributes for each vertex. Graphs were estimated using the NeuroData’s MR Graphs pipeline (23), with vertices representing subregions defined via spatial proximity and edges defined by tensor-based fiber streamlines connecting these regions (Fig. 2).

The actual graphs we consider are the largest connected component (LCC) of the induced subgraph on the vertices labeled as both left or right and gray or white. This yields m=114 connected graphs on n≈40,000 vertices. Additionally, for each graph every vertex has a {Left,Right} label and a {Gray,White} label, which we sometimes find convenient to consider as a single label in {LG,LW,RG,RW}.

### Sparsity.

The only notions of sparsity relevant here are linear algebraic: whether there are enough edges in the graph to support spectral embedding and whether there are few enough to allow for sparse matrix computations. We have a collection of observed connectomes and we want to cluster the vertices in these graphs, as opposed to in an unobserved sequence with the number of vertices tending to infinity. Our connectomes have, on average, n≈40,000 vertices and e≈2,000,000 edges, for an average degree 2e/n≈100 and a graph density e/n2≈0.0025.

### Synthetic Analysis.

**Fig. 3.**
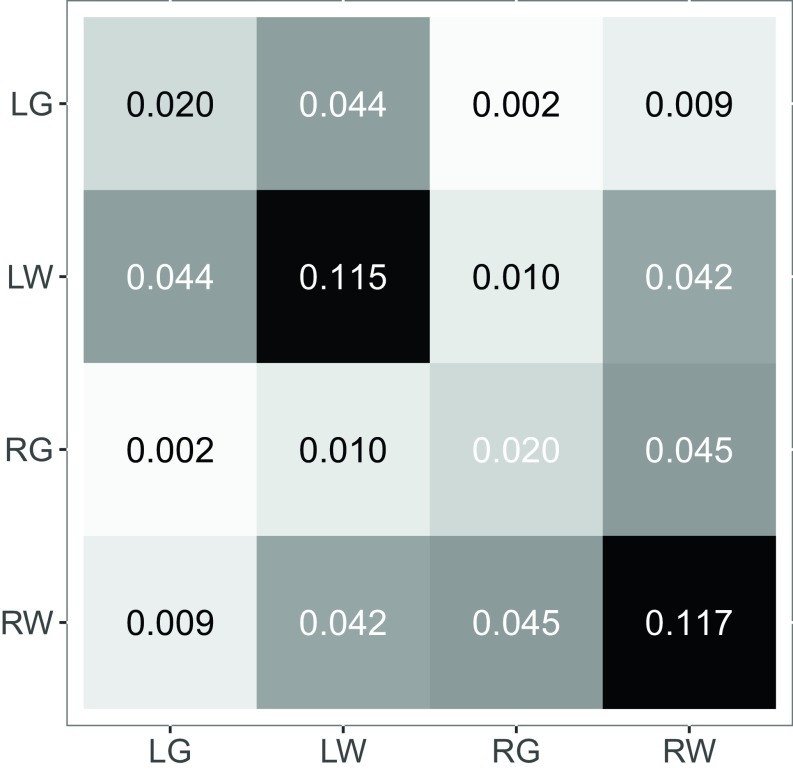
Block connectivity probability matrix for the {LG,LW,RG,RW} a priori projection of the composite connectome onto the four-block SBM. The two two-block projections ({Left,Right} and {Gray,White}) are shown in Fig. 4. This synthetic SBM exhibits the two-truths phenomenon both theoretically (via Chernoff analysis) and in simulation (via Monte Carlo).

We consider a synthetic data analysis via a priori projections onto the SBM—block model estimates based on known or assumed block memberships. Averaging the collection of m=114 connectomes yields the composite (weighted) graph adjacency matrix $\bar{A}$. The {LG,LW,RG,RW} projection of the binarized $\bar{A}$ onto the four-block SBM yields the block connectivity probability matrix B presented in Fig. 3 and the block membership probability vector $\vec{\pi }={\left[0.28,0.22,0.28,0.22\right]}^{⊤}$. Limit theory demonstrates that spectral graph clustering using d=K=4 will, for large n, correctly identify block memberships for this four-block case when using either LSE or ASE. Our interest is to compare and contrast the two spectral embedding methods for clustering into two clusters. We demonstrate that this synthetic case exhibits the two-truths phenomenon both theoretically and in simulation—the {LG,LW,RG,RW} a priori projection of our composite connectome yields a four-block two-truths SBM.

### Two-Block Projections.

**Fig. 4.**
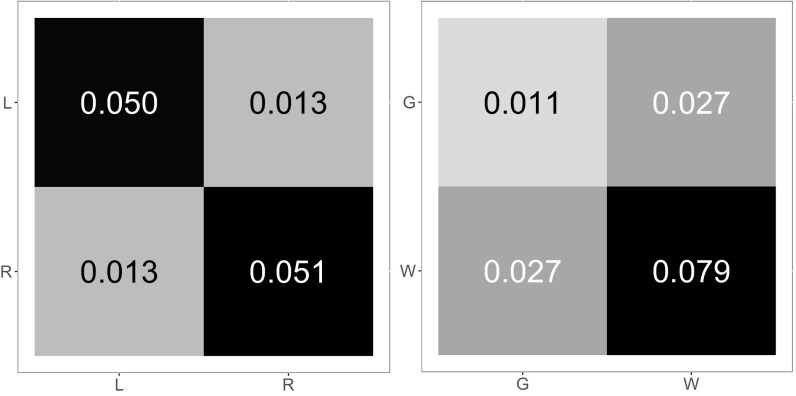
Block connectivity probability matrices for the a priori projection of the composite connectome onto the two-block SBM for (*Left*) {Left,Right} and (*Right*) {Gray,White}. {Left,Right} exhibits affinity structure, with Chernoff ratio <1; {Gray,White} exhibits core–periphery structure, with Chernoff ratio >1.

A priori projections onto the two-block SBM for {Left,Right} and {Gray,White} yield the two-block connectivity probability matrices shown in Fig. 4. It is apparent that the {Left,Right} a priori block connectivity probability matrix B=[a,b;b,c] represents an affinity SBM with a≈c≫b and the {Gray,White} a priori projection yields a core–periphery SBM with c≫a≈b. It remains to investigate the extent to which the Chernoff analysis from the two-block setting (LSE is preferred for affinity while ASE is preferred for core–periphery) extends to such a four-block two-truths case; we do so theoretically and in simulation using this synthetic model derived from the {LG,LW,RG,RW} a priori projection of our composite connectome in *Theoretical Results* and *Simulation Results* and then empirically on the original connectomes in *Connectome Results*.

### Theoretical Results.

Analysis using the large-sample Gaussian mixture model approximations from the LSE and ASE CLTs shows that the 2D embedding of the four-block model, when clustered into two clusters, will yield { {LG,LW}, {RG,RW} } (i.e., {Left, Right}) when embedding via LSE and { {LG,RG}, {LW,RW} } (i.e., {Gray, White}) when using ASE. That is, using numerical integration for the d=K=2 GMM ○ LSE, the largest Kullback–Leibler divergence (as a surrogate for Chernoff information) among the 10 possible ways of grouping the four Gaussians into two clusters is for the { {LG,LW}, {RG,RW} } grouping, and the largest of these values for the GMM ○ ASE is for the { {LG,RG}, {LW,RW} } grouping.

### Simulation Results.

We augment the Chernoff limit theory via Monte Carlo simulation, sampling graphs from the four-block model and running the GMM ○ {LSE,ASE} algorithm specifying $\hat{d}=\hat{K}=2$. This results in LSE finding {Left,Right} (ARI > 0.95) with probability >0.95 and ASE finding {Gray,White} (ARI > 0.95) with probability >0.95.

### Connectome Results.

**Fig. 5.**
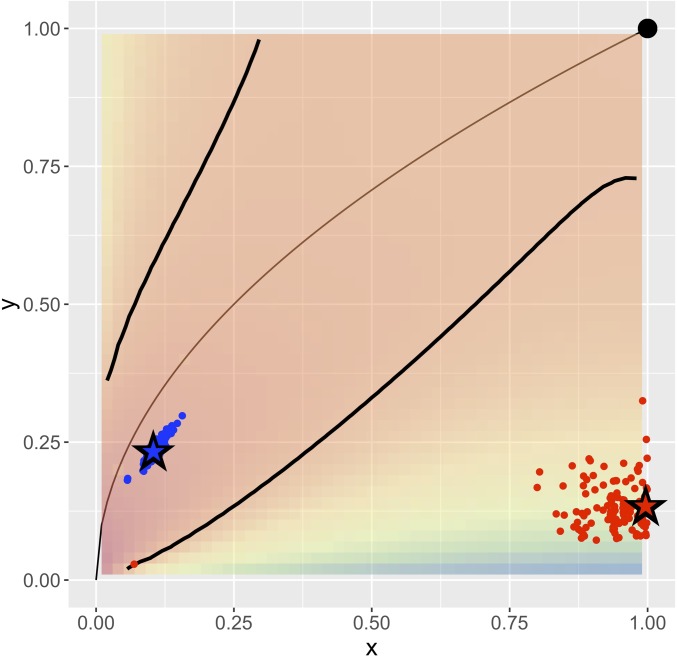
For each of our 114 connectomes, we plot the a priori two-block SBM projections for {Left,Right} in red and {Gray,White} in blue. The coordinates are given by x=min(a,c)/max(a,c) and y=b/max(a,c), where B=[a,b;b,c] is the observed block connectivity probability matrix. The thin black curve $y=\sqrt{x}$ represents the rank 1 submodel separating positive definite (lower right) from indefinite (upper left). The background color shading is Chernoff ratio ρ, and the thick black curves are ρ=1 separating the region where ASE is preferred (between the curves) from where LSE is preferred. The point (1,1) represents Erdős–Rényi (a=b=c). The large stars are from the a priori composite connectome projections (Fig. 4). We see that the red {Left,Right} projections are in the affinity region where ρ<1 and LSE is preferred while the blue {Gray,White} projections are in the core–periphery region where ρ>1 and ASE is preferred. This analytical finding based on projections onto the SBM carries over to empirical spectral clustering results on the individual connectomes (Fig. 7).

**Fig. 6.**
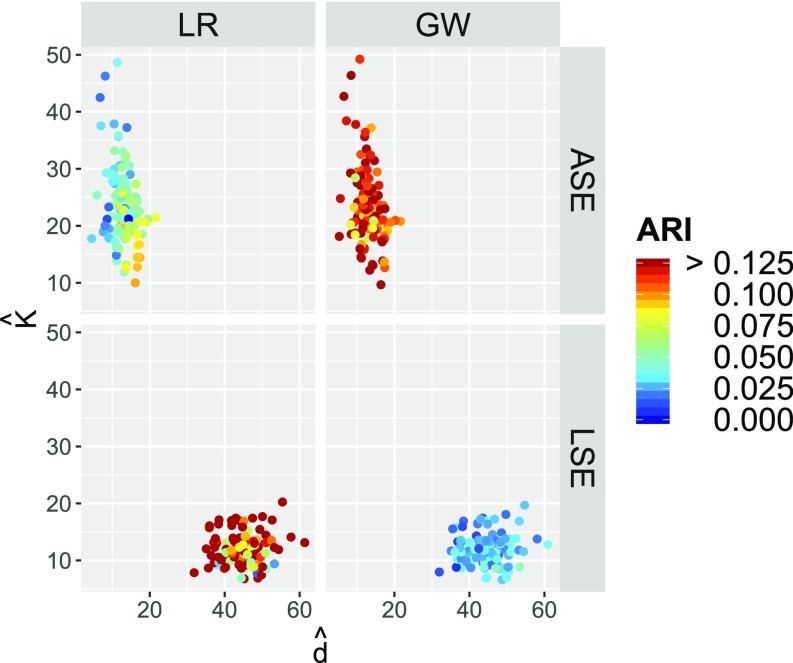
Results of the ($\hat{d},\hat{K}$) model selection for spectral graph clustering for each of our 114 connectomes. For LSE we see $\hat{d}\in \left\{30,\ldots ,60\right\}$ and $\hat{K}\in \left\{2,\ldots ,20\right\}$; for ASE we see $\hat{d}\in \left\{2,\ldots ,20\right\}$ and $\hat{K}\in \left\{10,\ldots ,50\right\}$. The color coding represents clustering performance in terms of ARI for each of LSE and ASE against each of the two truths {Left, Right} and {Gray, White} and shows that LSE clustering identifies {Left,Right} better than {Gray,White} and ASE identifies {Gray,White} better than {Left,Right}. Our two-truths phenomenon is conclusively demonstrated: LSE finds {Left,Right} (affinity) while ASE finds {Gray,White} (core–periphery).

**Fig. 7.**
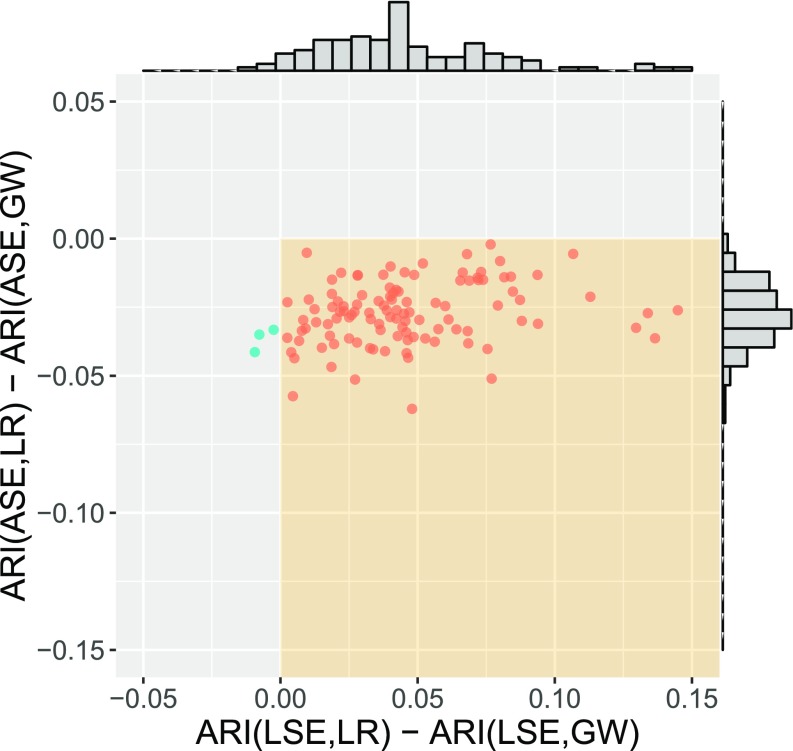
Spectral graph clustering assessment via ARI. For each of our 114 connectomes, we plot the difference in ARI for the {Left,Right} truth against the difference in ARI for the {Gray,White} truth for the clusterings produced by each of LSE and ASE: x = ARI(LSE,LR) – ARI(LSE,GW) vs. y = ARI(ASE,LR) – ARI(ASE,GW). A point in the (+,−) quadrant indicates that for that connectome the LSE clustering identified {Left,Right} better than {Gray,White} and ASE identified {Gray,White} better than {Left,Right}. Marginal histograms are provided. Our two-truths phenomenon is conclusively demonstrated: LSE identifies {Left,Right} (affinity) while ASE identifies {Gray,White} (core–periphery).

Figs. 5–7 present empirical results for the connectome dataset, m=114 graphs each on n≈40,000 vertices. We note that these connectomes are most assuredly not four-block two-truths SBMs of the kind presented in Figs. 3 and 4, but they do have two truths ({Left, Right} and {Gray, White}) and, as we shall see, they do exhibit a real-data version of the synthetic results presented above, in the spirit of semiparametric SBM fitting.

First, in Fig. 5, we consider a priori projections of the individual connectomes, analogous to the Fig. 4 projections of the composite connectome. Letting B=[a,b;b,c] be the observed block connectivity probability matrix for the a priori two-block SBM projection ({Left, Right} or {Gray, White}) of a given individual connectome, the coordinates in Fig. 5 are given by x=min(a,c)/max(a,c) and y=b/max(a,c). Each graph yields two points, one for each of {Left, Right} and {Gray, White}. We see that the {Left,Right} projections are in the affinity region (large x and small y imply a≈c≫b, where Chernoff ratio ρ<1 and LSE is preferred) while the {Gray,White} projections are in the core–periphery region [small x and small y imply max(a,c)≫b≈min(a,c), where ρ>1 and ASE is preferred]. This exploratory data analysis finding indicates complex two-truths structure in our connectome dataset. [Of independent interest, we propose Fig. 5 as the representative for an illustrative two-truths exploratory data analysis (EDA) plot for a dataset of m graphs with multiple categorical vertex labels.]

In Figs. 6 and 7 we present the results of m=114 runs of the spectral clustering algorithm GMM ○ {LSE,ASE}. We consider each of LSE and ASE, choosing $\hat{d}$ and $\hat{K}$ as described above. The resulting empirical clusterings are evaluated via the ARI against each of the {Left, Right} and {Gray, White} truths. In Fig. 6 we present the results of the ($\hat{d},\hat{K}$) model selection, and we observe that ASE is choosing $\hat{d}\in \left\{2,\ldots ,20\right\}$ and LSE is choosing $\hat{d}\in \left\{30,\ldots ,60\right\}$, while ASE is choosing $\hat{K}\in \left\{10,\ldots ,50\right\}$ and LSE is choosing $\hat{K}\in \left\{2,\ldots ,20\right\}$. In Fig. 7, each graph is represented by a single point, plotting x = ARI(LSE,LR) – ARI(LSE,GW) vs. y = ARI(ASE,LR) – ARI(ASE,GW), where “LSE” (resp. “ASE”) represents the empirical clustering $C_{LSE}$ (resp. $C_{ASE}$) and “LR” (resp. “GW”) represents the true clustering $C_{\left\{\text{Left,Right}\right\}}$ (resp. $C_{\left\{\text{Gray,White}\right\}}$). We see that almost all of the points lie in the (+,−) quadrant, indicating ARI(LSE,LR) > ARI(LSE,GW) and ARI(ASE,LR) < ARI(ASE,GW). That is, LSE finds the affinity {Left, Right} structure and ASE finds the core–periphery {Gray, White} structure. The two-truths structure in our connectome dataset illustrated in Fig. 5 leads to fundamentally different but equally meaningful LSE vs. ASE spectral clustering performance. This is our two-truths phenomenon in spectral graph clustering.

## Conclusion

The results presented herein demonstrate that practical spectral graph clustering exhibits a two-truths phenomenon with respect to Laplacian vs. adjacency spectral embedding. This phenomenon can be understood theoretically from the perspective of affinity vs. core–periphery stochastic block models and via consideration of the two a priori projections of a four-block two-truths SBM onto the two-block SBM. For connectomics, this phenomenon manifests itself via LSE better capturing the left hemisphere/right hemisphere affinity structure and ASE better capturing the gray matter/white matter core–periphery structure and suggests that a connectivity-based parcellation based on spectral clustering should consider both LSE and ASE, as the two spectral embedding approaches facilitate the identification of different and complementary connectivity-based clustering truths.
